# Targeting anticancer drug-induced senescence in glioblastoma therapy

**DOI:** 10.18632/oncotarget.26502

**Published:** 2018-12-25

**Authors:** Maja T. Tomicic, Markus Christmann

**Affiliations:** Markus Christmann: Department of Toxicology, University Medical Center Mainz, Mainz, Germany; Maja T. Tomicic: Department of Toxicology, University Medical Center Mainz, Mainz, Germany

**Keywords:** senescence, temozolomide, glioblastoma, senolytic drugs, cancer

Glioblastomas (WHO grade IV) account for 60-70% of high-grade brain tumours. Due to their aggressiveness, characterized by highly infiltrative growth, they remain incurable. Even using the optimum treatment protocol, namely maximum safe resection followed by radiotherapy with concomitant and/or adjuvant temozolomide (TMZ), less than 26.5% of patients survive for more than two years [1].

TMZ exerts its cytotoxic effect by the induction of O^6^MeG, and subsequently by the formation of DNA double-strand breaks (DSB), leading to the activation of cell death *via* apoptosis. However, as indicated by the poor response of glioma patients, the induction of apoptosis seems to be inefficient, at least at TMZ concentrations achievable in a tumour. Thus, we demonstrated that exposure of glioma cell lines to clinically achievable TMZ concentrations alone or in combination with ionizing radiation predominantly induces senescence and not apoptosis [2]. The high frequency of senescence could explain why exposure to TMZ/IR alone cannot be sufficient to kill glioma cells. TMZ-induced senescence was triggered by O^6^MeG and the activation of the ATR/CHK1 pathway. Concerning the molecular mechanism of senescence, we found that TMZ-induced cell cycle arrest occurs in the G2/M phase *via* CHK1-dependent CDC25c degradation and inactivation of the CyclinB/CDK1 complex. The G2/M arrest was further fixed into senescence by the activation of p21 [2]. Of note, TMZ-induced senescence seems to be independent of p14 and p16 but depends on p21 activation. Therefore, p53 deficient cells, which do not activate p21, only show the G2/M arrest, but not the senescence-specific phenotype(s).

TMZ-induced senescence was accompanied by abrogated (suppressed) DNA repair - mismatch repair and homologous recombination - due to the disruption of the E2F1/DP1 complex thereby leading to transcriptional silencing of the *EXO1, MSH2*, *MSH6* and *RAD51* genes [2]. The compromised DNA repair activity implicates that senescent cells may accumulate additional genomic alterations during ongoing TMZ/radiotherapy. In general, senescence is considered to permanently block proliferation. Several studies, however, have already indicated that cells can escape from genotoxin-induced senescence when the initial DNA damage stimuli are removed [3-5]. If this also holds true for TMZ exposed glioma cells, then these cells which accumulated genomic alterations during senescence could contribute to the formation of recurrences. We should stress that, by definition, senescence is irreversible. Therefore, if cells can indeed escape senescence, this type of cell cycle arrest, in which the cells show all known markers of senescence (ß-Gal positivity, SASP, SAHFs, SCARs,..) should rather be called “senescence-like dormant phase” (SLDP). However, the final proof for such an escape from anticancer drug-induced SLDP in tumour cells is still needed. Regardless of the name of this type of cell cycle arrest, selective killing of these cells is desirable for the following reasons. Firstly, it would prevent the potential formation of recurrences from these senescent cells, and secondly, it would reduce the negative physiological impact of the senescence-associated secretory phenotype (SASP).

Agents that specifically kill senescent cells are called “senolytics”. Their discovery was based on the observation that senescent cells are resistant to apoptosis, due to the upregulation of specific senescent-cell anti-apoptotic pathways (SCAPs) which might be caused by senescence-associated mitochondrial dysfunction (SAMD) (for review see [6, 7]). Senolytics, targeting these SCAPs in ageing and geriatric syndromes, have already been tested in several clinical trials. However, the use of senolytics could also enhance the effect of cancer chemotherapy [8, 9]. Among the already described senolytic agents, the BCL-2 inhibitor navitoclax has shown positive results *in vitro* and *in vivo*. Based on our experiments, upon TMZ exposure, not BCL-2 but rather proteins belonging to the class of IAPs seem to be important for protection against apoptosis [2] and, therefore, small-molecule inhibitors of these anti-apoptotic/pro-survival factors (IAP antagonists) should be tested as potential senolytics for TMZ-induced senescence.

Another important response to TMZ exposure is the activation of the SASP [2]. Generally, the SASP represents a clinically favourable response to chemotherapy because it might be involved in the maintenance of senescence and can additionally stimulate tumour clearance by the immune system. However, the SASP is a double-edged sword as it also exerts tumour-promoting activity [10]. Thus, SASP factors act as potent tumour promoters, enhancing malignant tumorigenesis. In detail, SASP can enhance the proliferation of neoplastic epithelial cells, promote the epithelial-to-mesenchymal transition (EMT) and tumour growth *in vivo*. Therefore, apart from senolytics, “anti-SASP therapy” has also been proposed for the improvement of chemotherapy (for review see [10]). However, also in this case, most studies addressing the effect of such therapy have been performed in the field of geriatric medicine. Whether such an approach could have a positive impact on TMZ-based glioma therapy or an unfavourable impact due to a reduced immune response has to be evaluated.

**Figure 1:**
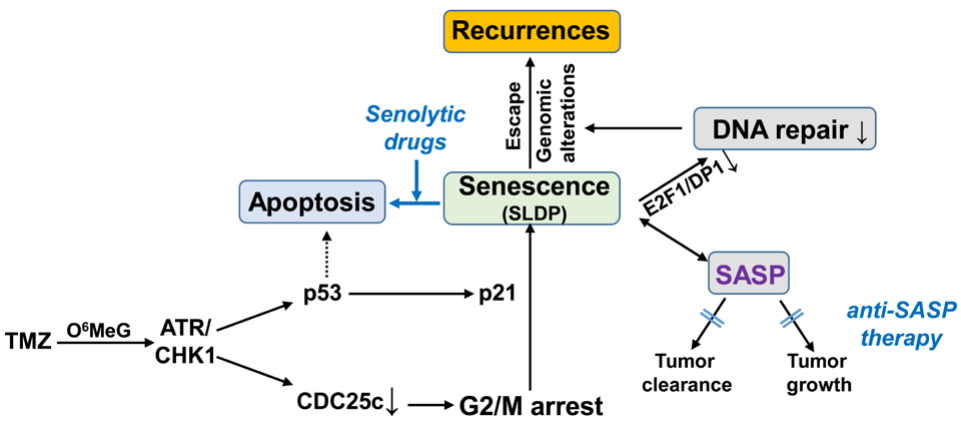
The potential impact of senolytic drugs and anti-SASP therapy on TMZ-induced anticancer therapy of gliomas

In summary, it is quite obvious by the poor therapeutic response of glioblastomas that the current TMZ-based therapy alone is not sufficient. As shown in our study, TMZ is rather weak at killing glioma cells, but at the same time efficiently induces senescence [2]. Since senescent cells may represent a threat for a patient due to the SASP or due to the potential senescence evasion leading to increased aggressiveness, targeting of senescent cells during or after TMZ therapy could be beneficial. For these reasons, further studies in this direction (schematically highlighted in Figure 1) are urgently needed.
